# Gut microbiota promotes cholesterol gallstone formation by modulating bile acid composition and biliary cholesterol secretion

**DOI:** 10.1038/s41467-021-27758-8

**Published:** 2022-01-11

**Authors:** Hai Hu, Wentao Shao, Qian Liu, Ning Liu, Qihan Wang, Jin Xu, Xin Zhang, Zhenkun Weng, Qifan Lu, Long Jiao, Chaobo Chen, Haidong Sun, Zhaoyan Jiang, Xiaoping Zhang, Aihua Gu

**Affiliations:** 1 Center of Gallbladder Disease, Shanghai East Hospital, Institute of Gallstone Disease, Tongji University School of Medicine, Shanghai, China; 2 State Key Laboratory of Reproductive Medicine, School of Public Health, Nanjing Medical University, Nanjing, Jiangsu China; 3 Collaborative Innovation Center for Cardiovascular Disease Translational Medicine, Center for Global Health, Nanjing Medical University, Nanjing, Jiangsu China; 4 School of Instrument Science and Engineering, Southeast University, Nanjing, Jiangsu China; 5 State Key Laboratory of Reproductive Medicine (Suzhou Center), Gusu School, Nanjing Medical University, Suzhou, Jiangsu China; 6 School of Electronic Information and Electrical Engineering, Shanghai Jiao Tong University, Shanghai, China; 7 Department of Institution of Interventional and Vascular Surgery, Tongji University School of Medicine, Shanghai, China

**Keywords:** Bacterial pathogenesis, Cholelithiasis, Microbiome

## Abstract

Cholesterol gallstone disease is a worldwide common disease. Cholesterol supersaturation in gallbladder bile is the prerequisite for its pathogenesis, while the mechanism is not completely understood. In this study, we find enrichment of gut microbiota (especially *Desulfovibrionales)* in patients with gallstone disease. Fecal transplantation of gut microbiota from gallstone patients to gallstone-resistant strain of mice can induce gallstone formation. Carrying *Desulfovibrionales* is associated with enhanced cecal secondary bile acids production and increase of bile acid hydrophobicity facilitating intestinal cholesterol absorption. Meanwhile, the metabolic product of *Desulfovibrionales*, H_2_S increase and is shown to induce hepatic FXR and inhibit CYP7A1 expression. Mice carrying *Desulfovibrionales* present induction of hepatic expression of cholesterol transporters *Abcg5/g8* to promote biliary secretion of cholesterol as well. Our study demonstrates the role of gut microbiota, *Desulfovibrionales*, as an environmental regulator contributing to gallstone formation through its influence on bile acid and cholesterol metabolism.

## Abstract (web summary)

Metabolic conditions associated with alterations of the gut microbiome, such as obesity and diabetes, predispose to gallstone disease. Here the authors demonstrate that the gut microbiome, in particular the genus Desulfovibrionale, contribute to gallstone formation in mice.

## Introduction

Cholesterol gallstone disease (GS) is a worldwide prevalent disease, especially in western countries^1^. Our recent survey found its incidence to be over 12% in Shanghai, China. The prerequisite biochemical disorder leading to gallstone is the formation of cholesterol supersaturated bile in the gallbladder. It is known that both genetic inclination and environmental factors contribute to the pathogenesis of this disease, while the mechanism is not completely understood^2^. Different strains of mice have different susceptibility to gallstone formation^3^. Phenotypic changes differed during the process of gallstone formation under lithogenic diet (LD) between strains^4,5^. In humans, gallstone is prevalent in certain ethnicities, such as Pima Indians and certain pedigrees. The Swedish Twin study demonstrated a heritability of 25% for GS^6^. On the other hand, non-genetic risk factors as metabolic disorders (i.e., obesity, diabetes mellitus, non-alcoholic fatty liver disease, etc.), were highly connected with gallstone formation^7–9^.

In recent years, the gut microbiota has been recognized to be contributors to metabolic disorders^10^ such as diabetes mellitus^11^, obesity^12^, fatty liver disease^13^, atherosclerosis^14^, and others^15^. The studies also provided evidence that metabolites including short-chain fatty acids, amino acids, produced by the gut microbiota exerted regulatory roles in remote organs as the liver^16^. These results led us to hypotheses that gut microbiota might play an important role in influencing host gallstone formation. The first clue of the possible involvement of bacteria in gallstone formation could be dated back to an early study decades ago^17^, in which they showed less incidence of gallstone in germ-free mice. Another study demonstrated that in mice infected with enterohepatic *Helicobacter* spp. could promote cholesterol gallstones^18^. However, there is a lack of enough evidence to explore the mechanisms for gut microbiota contributing to gallstone formation.

In this study, we identified enrichment of *Desulfovibrionale* in feces from patients with cholesterol GS as well as in gallstone-susceptible mice. By fecal microbiota transplantation (FMT) from gallstone patients or by co-housing with gallstone-susceptible mice, gallstone formation could be induced in gallstone-resistance mice. We further demonstrated that carrying *Desulfovibrionale* was related to an increase of secondary bile acid production due to activated bile acid dehydroxylation of gut microbiota, leading to an increase of bile acid hydrophobicity which facilitated intestinal cholesterol absorption, resulting in hepatic accumulation of cholesterol and enhancement of biliary cholesterol secretion.

## Results

### Enrichment of *Desulfovibrionales* in patients with cholesterol GS

**Fig. 1:**
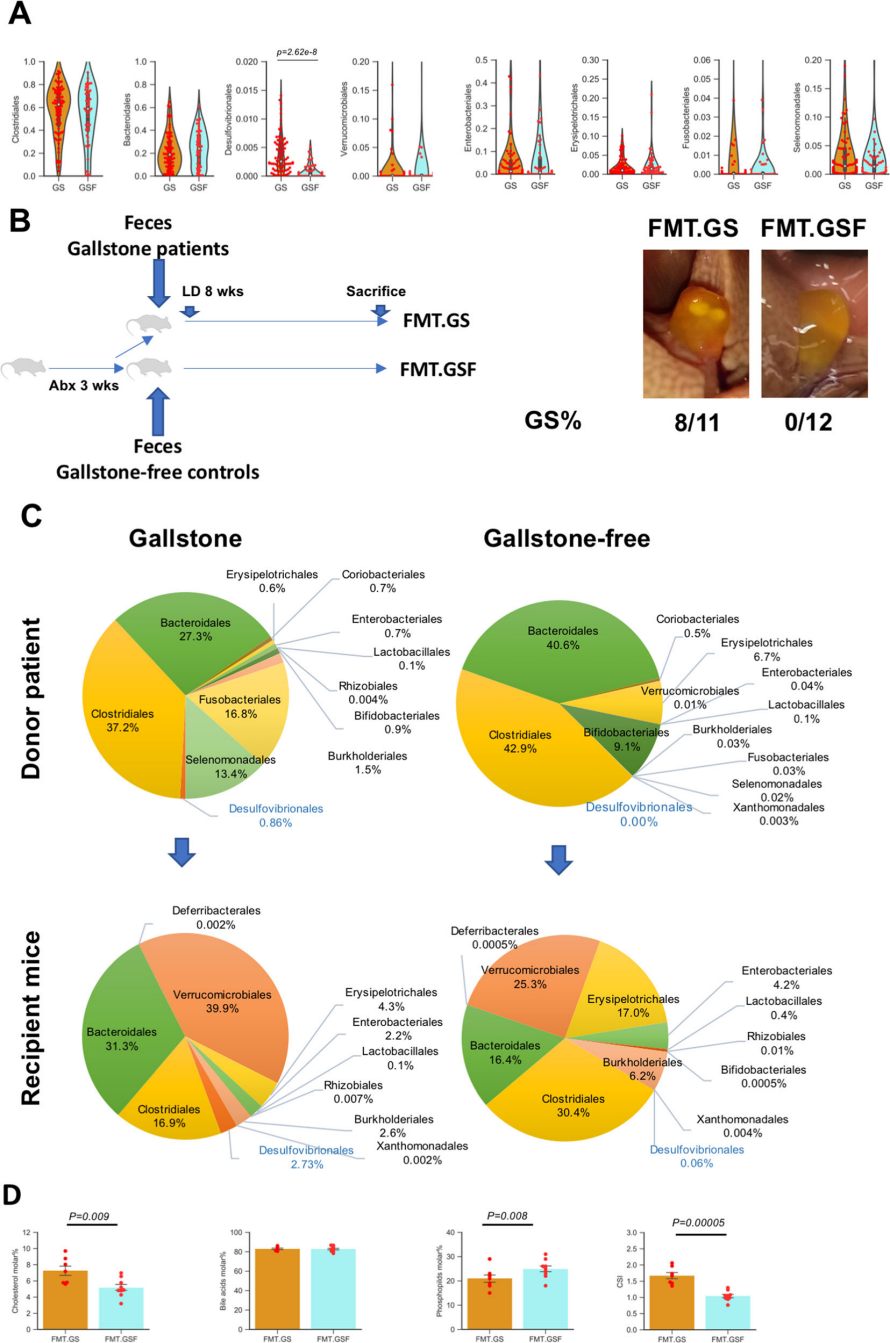
Fecal microbiota transplantation induced gallstone formation. **A** Violin plot showed the abundance of gut microbiota at the order level in feces from patients with cholesterol gallstone disease (GS: *n* = 80) and gallstone-free controls (GSF, *n* = 49). Statistics was performed by a two-sided *t* test. **B** Left: Schematic shows the process of human fecal microbiota transplantation to mice. Right: Incidence of gallstone in gallstone-resistant AKR/J mice receiving transplantation of feces from patients with gallstone (“FMT.GS”: *n* = 11 mice) and gallstone-free controls (“FMT.GSF”: *n* = 12 mice). **C** Abundance of gut microbiota at the order level in feces from donor patients and cecal content from recipient mice. **D** Biliary lipid composition and cholesterol saturation index (CSI) in gallbladder bile from FMT.GS mice (*n* = 7) and FMT.GSF mice (*n* = 9). Data are expressed as mean ± SEM. Statistics was performed by a two-sided *t* test. Source data are provided as a Source data file.

Feces samples from 80 patients with cholesterol GS and 49 gallstone-free controls (GSF) were collected. The fecal microbiota composition of both groups was analyzed by 16s rRNA sequencing. The microbiota composition showed a higher abundance of *Desulfovibrionales* order in GS patients than in GSF controls (Fig. 1A), which suggested an association between *Desulfovibrionales* and gallstone susceptibility.

### *Desulfovibrionales* enriched human FMT induced gallstone formation in gallstone-resistant mice

Donor feces enriched in *Desulfovibrionales* from gallstone patients (GS) or feces from gallstone-free (GSF) controls were transplanted to gallstone-resistant AKR/J mice (GS-R mice). No gallstone formed in mice subjected to transplantation of feces from GSF controls (0/12) after 8-week LD. *Desulfovibrionales* were not detected in these mice. However, gallstone formed in 73% (8/11) of the mice receiving feces from the GS donors (Fig. 1B). The profiles of gut microbiota suggested the successful introduction of *Desulfovibrionales* to these mice (Fig. 1C). Biliary composition analysis showed the increased cholesterol molar and cholesterol saturation index (CSI) (Fig. 1D) in the gallbladder bile in mice carrying *Desulfovibrionales*. Serum high-density lipoprotein, low-density lipoprotein cholesterol (Fig. S1A), and hepatic cholesterol (Fig. S1B) levels were higher as well. The results demonstrated that *Desulfovibrionales* from gallstone patients were capable to induce gallstone formation.

### Introducing *Desulfovibrionales* to gallstone-resistant mice by co-housing with gallstone susceptible-mice promoted gallstone formation

**Fig. 2:**
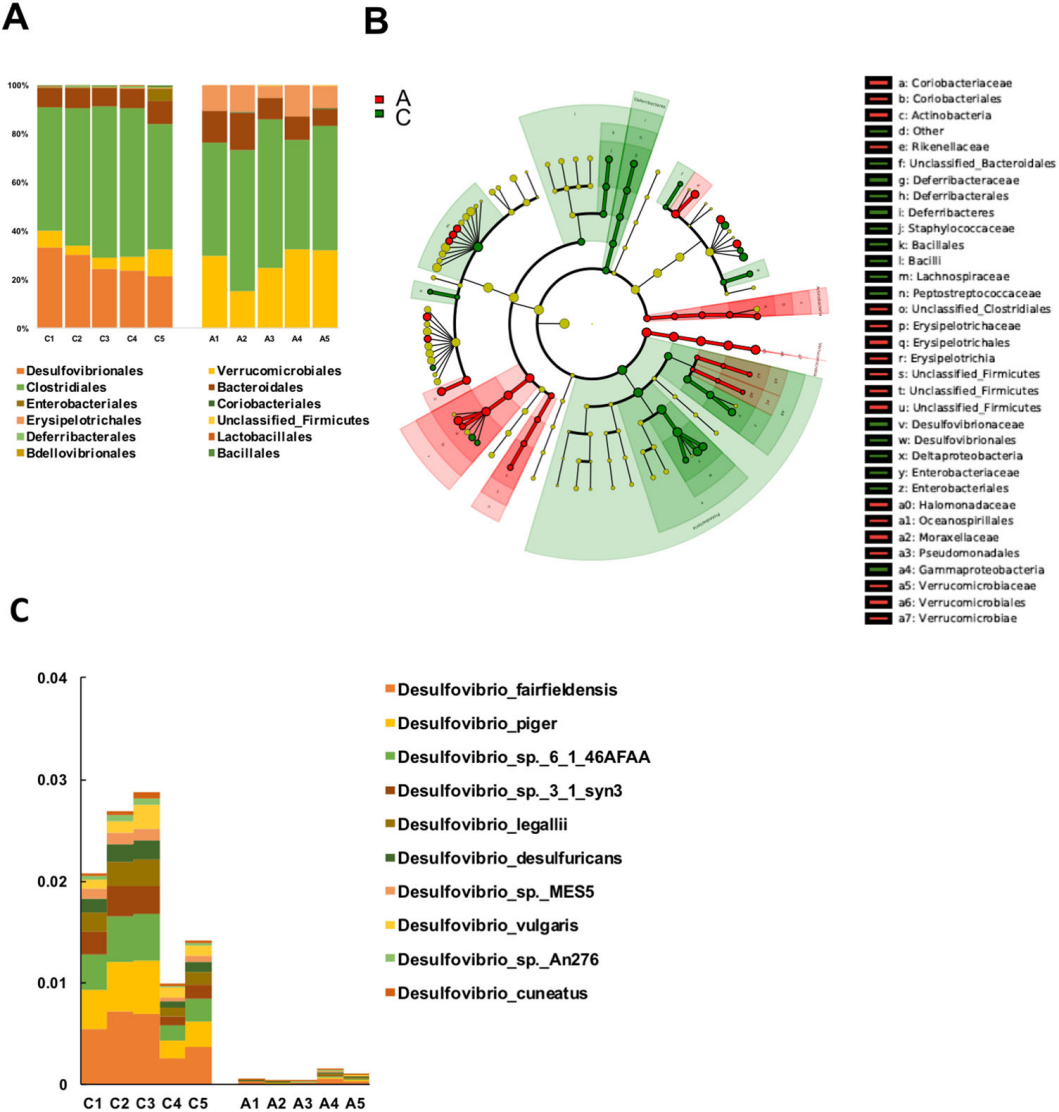
Enrichment of Desulfovibrionales in association with gallstone disease in mice. **A** Enrichment of *Desulfovibrionales* in C57BL/6J mice than AKR/J mice fed with the lithogenic diet for 8 weeks (*n* = 5 mice/group). **B** LEfSe cladogram showed the different abundance of microbiota between strains of mice. The diameter of each circle was proportional to its abundance. **C** Abundance of *Desulfovibrio* species in each group of mice. “C” represented gallstone-susceptible mice C57BL/6J strain and “A” represented gallstone-resistant mice AKR/J strain. (*n* = 5 mice/group). Source data are provided as a Source data file.

Comparing the gut microbiota between the gallstone-susceptible mice C57BL/6J (GS-S mice), and the gallstone-resistant mice AKR/J (GS-R mice), we found that *Desulfovibrionales* order was also markedly abundant in C57BL/6J mice (Fig. 2A) as well as shown by LEfSe algorithm (Fig. 2B and Fig. S2)*. Desulfovibrio fairfieldensis*, *piger*, *desulfuricans*, and *vulgaris* were identified to be the major species (Fig. 2C) in GS-S mice, which were barely detected in GS-R mice.

**Fig. 3:**
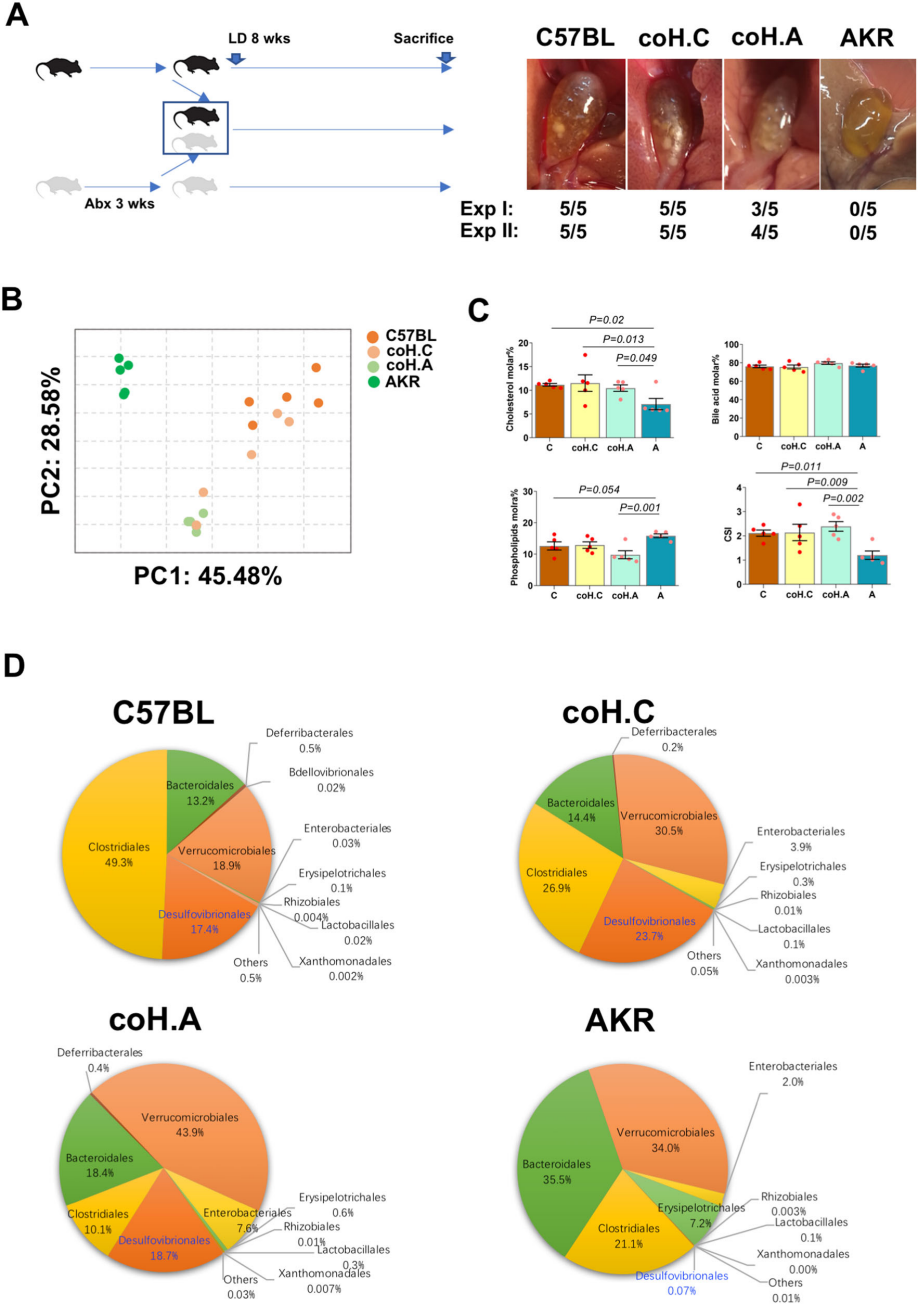
Co-housing promoted gallstone formation in gallstone-resistant mice. **A** Left: a schematic show of the process of co-housing experiment. Right: incidence of gallstone in each group of mice (*n* = 10 mice/group). **B** Principal coordinate analysis of gut microbiota in cecum contents from each group of mice. **C** Biliary lipid composition and cholesterol saturation index (CSI) of gallbladder bile in each group (*n* = 5 mice/group). Data are expressed as mean ± SEM. Statistics was performed by two-sided ANOVA with LSD post hoc analysis between groups. **D** Abundance of gut microbiota at the order level in each group (*n* = 5 mice/group). “C”: C57BL/6J, “A”: AKR/J, “coH.C”: C57BL/6J co-housing with AKR/J and “coH.A”: AKR/J co-housing with C57BL/6J. Source data are provided as a Source data file.

Co-housing (Fig. 3A) successfully modified the gut microbiota in GS-R mice from the original mice (Fig. 3B) with significant enrichment of *Desulfovibrionales* order in the co-housing GS-R mice (Fig. 3D). As a result, gallstone formation increased to 70% in co-housing GS-R mice (Fig. 3A). Profiles of gallbladder bile composition from co-housing GS-R mice turned to resemble GS-S mice, presenting as elevated biliary cholesterol molar percentage and CSI (Fig. 3C), a condition favoring gallstone formation.

### Carrying *Desulfovibrionales* influenced bile acid profiles

**Fig. 4:**
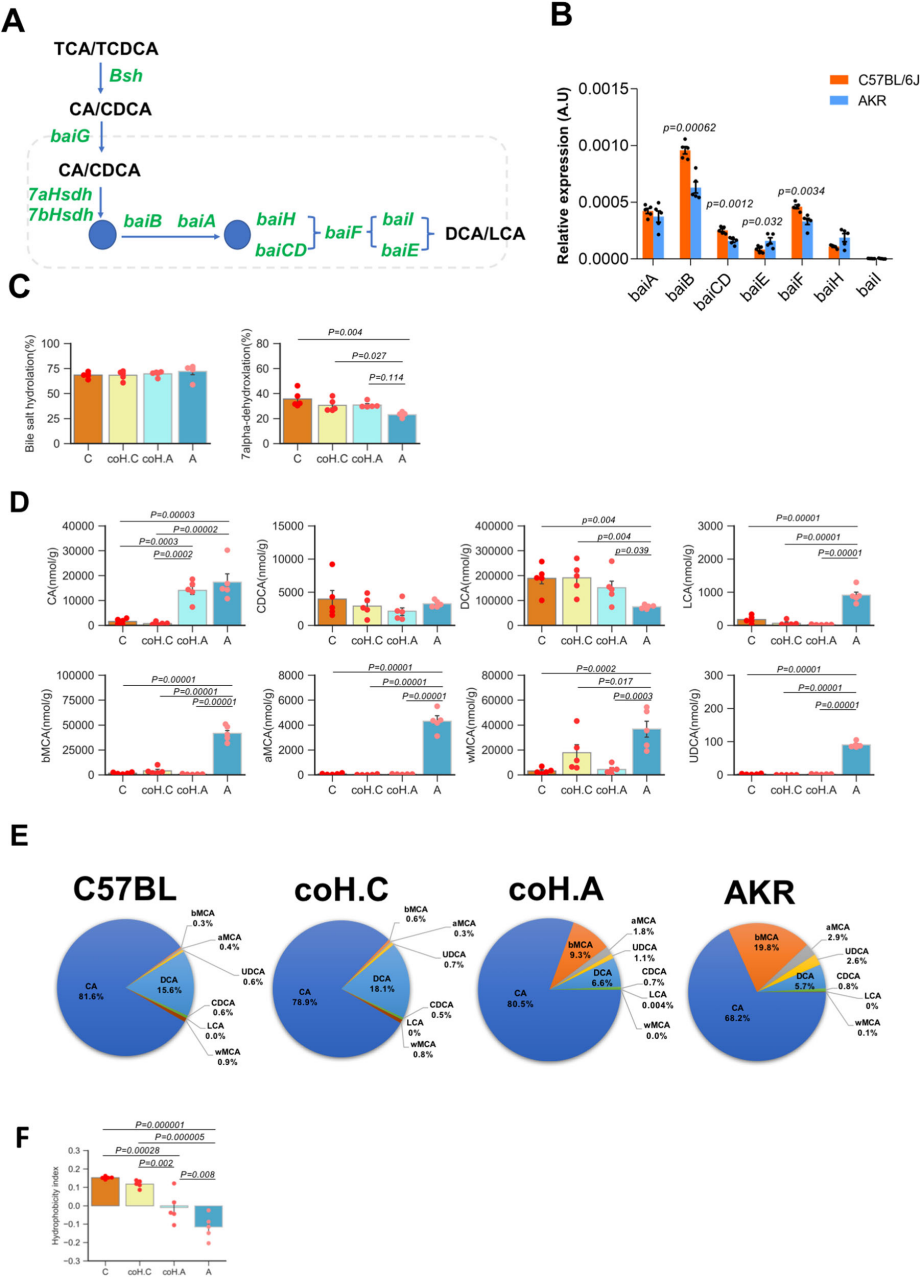
Changes in bile acid metabolism in gallstone-resistant mice after co-housing. **A** Schematics of the bacterial BA biotransformation pathways of deconjugation and multiple steps of 7α/β-dehydroxylation. **B** Comparison of the relative abundances of genes encoding enzymes involved in bacterial bile acid metabolism between gallstone-susceptible mice C57BL/6J and gallstone-resistant mice AKR/J (*n* = 5 mice/group). Data are expressed as mean ± SEM. Statistics was performed by a two-sided *t* test. **C** Comparison of bacterial bile salt hydrolase and 7α-dehydroxylation activities in cecum contents from groups of mice (*n* = 5 mice/group). Data are expressed as mean ± SEM. Statistics was performed by two-sided ANOVA with LSD post hoc analysis between groups. **D** Cecal bile acid compositions in each group of mice (*n* = 5 mice/group). Data are expressed as mean ± SEM. Statistics was performed by two-sided ANOVA with LSD post hoc analysis between groups. **E** Composition of bile acids in gallbladder bile from each group of mice. **F** Hydrophobicity index (HI) of bile acids in gallbladder bile in each group (*n* = 5 mice/group). Data are expressed as mean ± SEM. Statistics was performed by two-sided ANOVA with LSD post hoc analysis between groups. “C”: C57BL/6J, “A”: AKR/J, “coH.C”: C57BL/6J co-housing with AKR/J and “coH.A”: AKR/J co-housing with C57BL/6J. Source data are provided as a Source data file.

A major role of *Desulfovibrionales* is to reduce substrates such as taurine into H_2_S, which was reported to be a necessary grow factor for 7α-dehydroxylating bacteria^19^. Interestingly, comparing the multi-gene BA-inducible (*Bai*) operon encoding genes involved in 7-α/β dehydroxylation of BAs (Fig. 4A), we found that GS-S mice had higher expression of *BaiB*, *CD*, and *F* (Fig. 4B), indicating an active bile acid dehydroxylation in these mice. Accordingly, 7α-dehydroxylation activity in the cecum content was significantly higher in GS-S mice as well as GS-R mice after co-housing (Fig. 4C).

Next, we measured the cecal bile acids. As expected, DCA, the product of bacteria 7a-dehydroxylation and the major component of cecal bile acids, was significantly higher in GS-S mice and co-housing GS-R mice (Fig. 4D).

In gallbladder bile, cholic acid (CA) and DCA, comprised >97% of the total bile acids in GS-S mice (Fig. 4E and Fig. S3A–C). However, GS-R mice retained a considerable amount of hydrophilic bile acids: β-muricholic acid (βMCA), αMCA, and ursodeoxycholic acid (UDCA) in their bile (≈25%), but less hydrophobic DCA. Co-housing led to a 50% decrease of βMCA and an 18% increase of CA in GS-R mice (Fig. 4E and Fig. S3C). The hydrophobicity index (HI) of bile acids was significantly higher in GS-S mice and co-housing GS-R mice (Fig. 4F).

Furthermore, GS-R mice receiving FMT from the gallstone patients also had higher cecal 7α-dehydroxylation activity (Fig. S4A), indicating a similar enhancement of secondary bile acids conversion. In line with this, DCA and ωMCA were higher, but βMCA, αMCA, and UDCA were lower in gallbladder bile (Fig. S4B, C), leading to higher HI in these mice (Fig. S4D).

These data collectively indicated that introducing *Desulfovibrionales* to GS-R mice led the bile acid profile to be more hydrophobic.

### Influences on hepatic transcripts of bile acid and cholesterol metabolism in mice carrying *Desulfovibrionales*

**Fig. 5:**
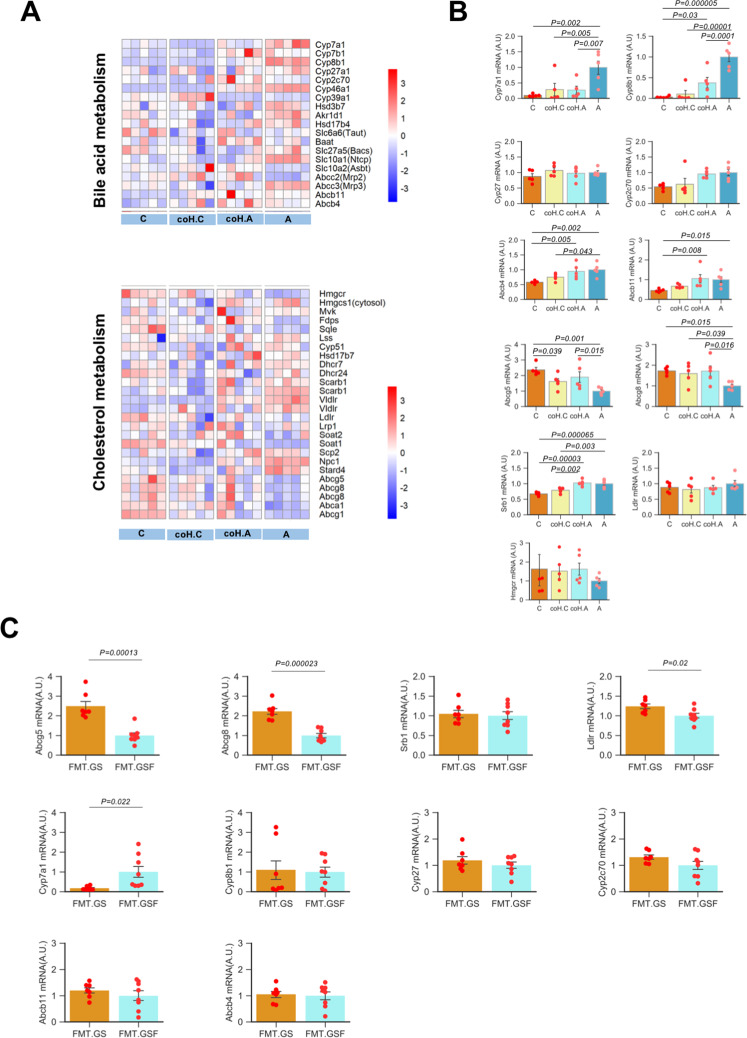
Influences on transcripts involved in hepatic cholesterol and bile acid metabolism in gallstone-resistant mice carrying *Desulfovibrionales*. **A** Heatmap shows mRNA expression levels of genes involved in hepatic bile acid and cholesterol metabolism from RNA-seq data in liver samples from C57BL/6J, AKR/J, and co-housing mice (*n* = 5 mice/group). “C”: C57BL/6J, “A”: AKR/J, “coH.C”: C57BL/6J co-housing with AKR/J and “coH.A”: AKR/J co-housing with C57BL/6J. **B** mRNA expression levels of key enzymes in hepatic bile acid synthesis and secretion, cholesterol synthesis, absorption, and secretion determined by quantitative real-time PCR (*n* = 5 mice/group). Data are expressed as mean ± SEM. Statistics was performed by two-sided ANOVA with LSD post hoc analysis between groups. **C** Quantitative real-time PCR analysis of mRNA expression of genes in hepatic bile acid and cholesterol metabolism in mice receiving fecal microbiota transplantation (FMT) from gallstone patient (FMT.GS, *n* = 7) or gallstone-free controls (FMT.GSF, *n* = 9). Data are expressed as mean ± SEM. Statistics was performed by a two-sided *t* test. Source data are provided as a Source data file.

To further unravel the impacts of *Desulfovibrionale* on the hepatic metabolic disorders in promoting gallstone formation, we performed RNA-seq in liver samples from mice in the co-housing experiment (Fig. 5A). Co-housing could further repress the expression of genes related to bile acid synthesis in GS-R mice, especially the expression of the rate-limiting enzymes of bile acid synthesis—cholesterol 7α-hydroxylase (*Cyp7a1*), the enzyme responsible for cholic acid synthesis—*Cyp8b1*. qRT-PCR further validated the inhibition of their expression in AKR/J mice after co-housing (Fig. 5B).

The gene expression profile in hepatic cholesterol metabolism was also changed (Fig. 5A). 3α-hydroxy-3-methyl-glutarylcoenzeme A (*Hmgcr*), the rate-limiting enzyme for cholesterol synthesis, was higher in GS-S mice even under conditions when cholesterol was overloading (Fig. 5A, B). The expression of *Hmgcr* elevated in GS-R mice after co-housing. The expressions of hepatic cholesterol transporters, ATP binding cassette (*Abc*) *g5* and *Abcg8*, were elevated GS-R mice after co-housing (Fig. 5A, B).

In the GS-R mice receiving FMT from gallstone patients, similar changes of genes in bile acid and cholesterol metabolism could be observed after carrying *Desulfovibrionales* (Fig. 5C).

### The product of *Desulfovibronales* regulated hepatic FXR-CYP71 expression

**Fig. 6:**
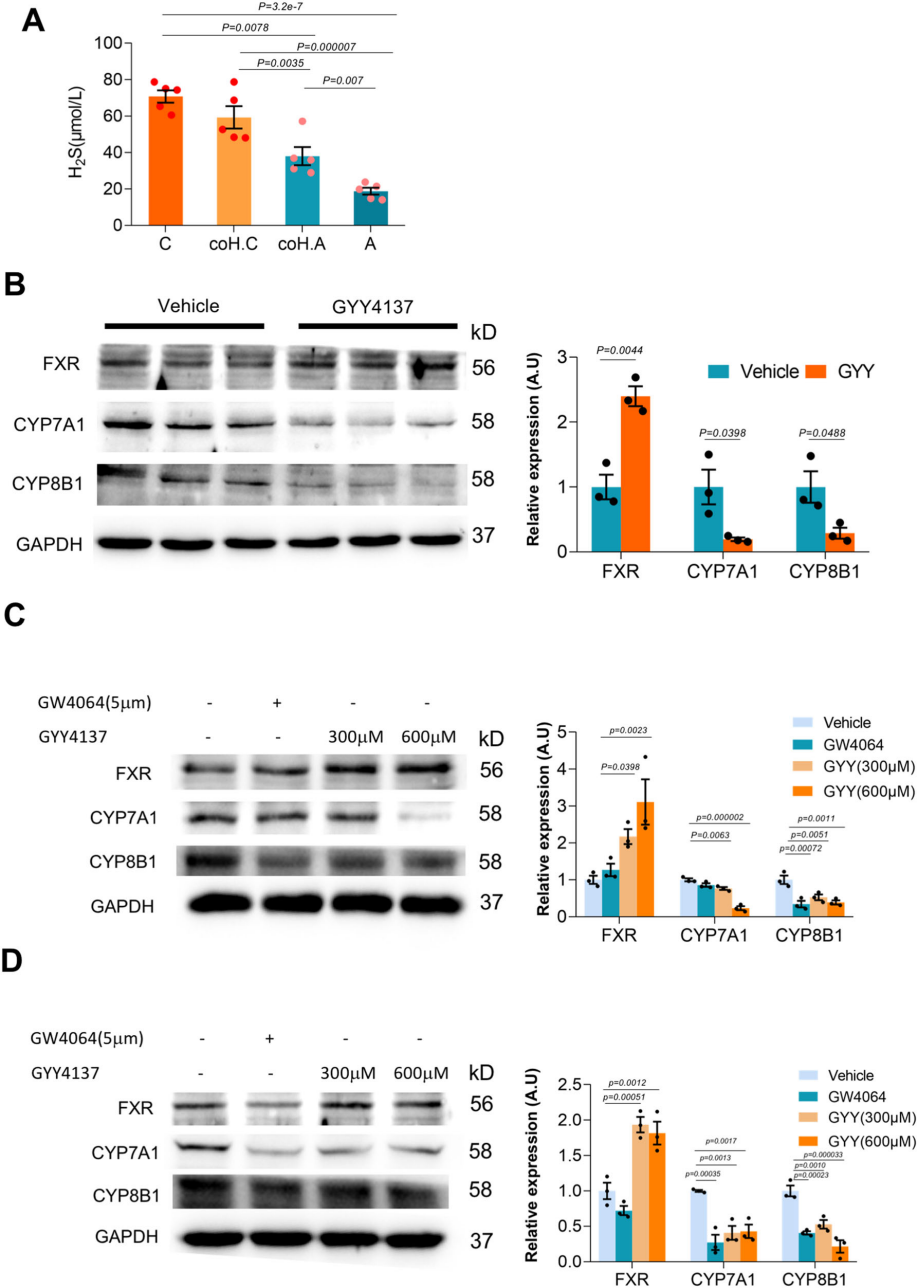
Regulation of hepatic FXR-CYP7A pathway by H2S. **A** Serum levels of H_2_S in co-housing mice (*n* = 5 mice/group). Data are expressed as mean ± SEM. Statistics was performed by two-sided ANOVA with LSD post hoc analysis between groups. “C”: C57BL/6J, “A”: AKR/J, “coH.C”: C57BL/6J co-housing with AKR/J and “coH.A”: AKR/J co-housing with C57BL/6J. **B** GYY4137 induced FXR protein and inhibited CYP7A1 protein expression in mouse liver (*n* = 3 mice/group). Data are expressed as mean ± SEM. Statistics was performed by a two-sided *t* test. **C** Regulation of FXR and CYP7A1 by GYY4137 in mouse hepatocytes Hepa1-6 (3 independent experiments/treatment). Data are expressed as mean ± SEM. Statistics was performed by two-sided ANOVA with LSD post hoc analysis between groups. **D** Regulation of FXR and CYP7A1 by GYY4137 in human hepatoma cells HuH7 (three independent experiments/treatment). Data are expressed as mean ± SEM. Statistics was performed by two-sided ANOVA with LSD post hoc analysis between groups. Source data are provided as a Source data file.

*Desulfovibrionales* contains genes for sulfate reduction to reduce sulfate to H_2_S. In GS-S mice (C57BL/6J) had significantly higher serum H_2_S than GS-R mice (AKR/J) and co-housing led to an increase of serum H_2_S in GS-R mice (Fig. 6A). H_2_S was reported to enhance FXR expression in HepG2 cells^20^, which in turn inhibited CYP7A1 expression^21^. To collaborate this, we treated mice with GYY4137, a novel H_2_S donor releasing H_2_S slowly and mimicking the time course of H_2_S release in vivo^22^. We found GYY4137 increased FXR protein and decreased CYP7A1 protein in liver tissues (Fig. 6B). Furthermore, both in the murine hepatocyte, Hepa1-6 (Fig. 6C), and human hepatoma cells, HuH7 (Fig. 6D), a dose-dependent induction of FXR and inhibition of CYP7A1 expression was found. These data suggested the existence of a regulatory pathway of hepatic bile acid metabolism by *Desulfobibronale* through its product H_2_S.

### Species of *Desulfovibro* promoted gallstone formation in mice

**Fig. 7:**
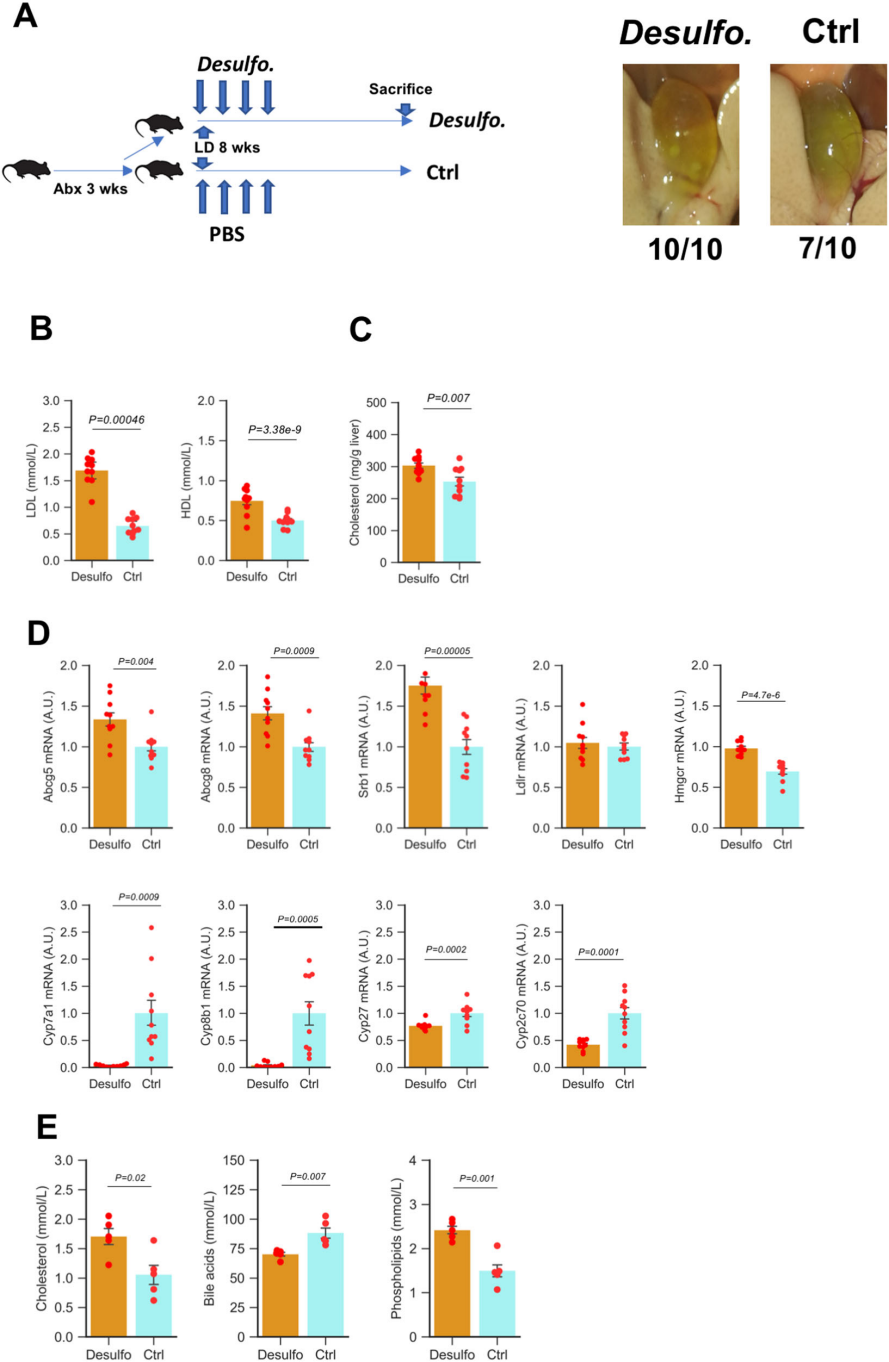
Species of *Desulfovibro* promoted gallstone formation in mice. **A** Incidence of gallstone (*n* = 10 mice/group). **B** Serum cholesterol in high-density lipoprotein (HDL) and low-density lipoprotein (LDL) (*n* = 10 mice/group). Data are expressed as mean ± SEM. Statistics was performed by a two-sided *t* test and **C** hepatic cholesterol levels (*n* = 10 mice/group). Data are expressed as mean ± SEM. Statistics was performed by a two-sided *t* test. **D** Quantitative real-time PCR analysis of gene expression levels in hepatic cholesterol and bile acid metabolism (*n* = 10 mice/group). Data are expressed as mean ± SEM. Statistics was performed by a two-sided *t* test. **E** Lipid composition in hepatic bile collected by common bile duct cannulation in each group (*n* = 5 mice/group). Data are expressed as mean ± SEM. Statistics was performed by *t* test. “*Desulfo*”: antibiotics-pre-treated C57BL/6J mice receiving *Desulfovibrio* species. “Ctrl”: antibiotics-pre-treated control C57BL/6J mice. Source data are provided as a Source data file.

To further validate the role of *Desulfovibrio* species on gallstone formation, we transplanted three commercially available species of *Desulfovibrio piger*, *desulfuricans*, and *vulgaris* to antibiotics-pre-treated GS-S mice. Depletion of gut microbiota decreased the gallstone incidence to 70% (7/10). In contrast, *Desulfovibrio* species transplantation restored gallstone incidence to 100% (10/10, Fig. 7A). Similarly, *Desulfovibrio* species transplantation could induce gallstone formation in 42% (5/12) of the GS-R mice (Fig. S5A), whereas none of the control GS-R mice formed gallstone (0%).

In the *Desulfovibrio* species transplantation group of mice, serum and hepatic cholesterol levels were significantly elevated in the transplanted mice (Fig. 7B, C). Moreover, expressions of genes in hepatic bile acid synthesis, *Cyp7a1, Cyp8b1, Cyp27,* and *Cyp2c70* were inhibited, and the expressions of *Abcg5, Abcg8, Srb1,* and *Hmgcr* were elevated (Fig. 7D and Fig. S5B).

To monitor the influence of *Desulfovibrio* species on the hepatic secretion of biliary lipids, hepatic bile was collected by common bile duct cannulation. In mice with *Desulfovibrio* species transplantation, a significant increase of cholesterol and phospholipids but less bile acid output in their hepatic bile was observed (Fig. 7E). This result suggested that carrying *Desulfovibrio* species promoted the hepatic secretion of cholesterol into bile.

## Discussion

**Fig. 8:**
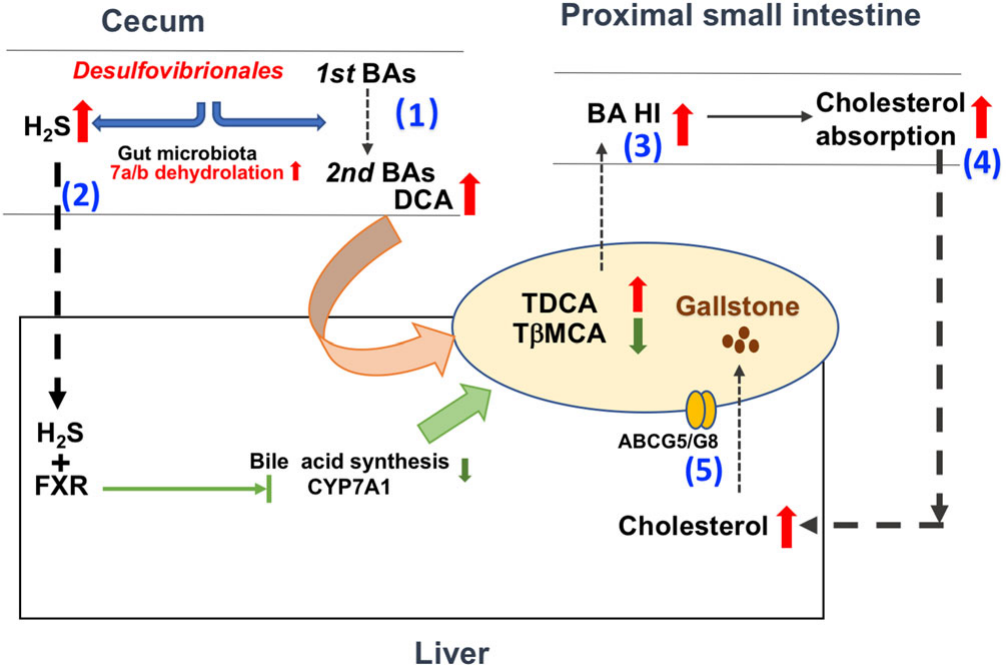
Schematic shows the underlying mechanisms of how gallstone-prone microbiota promotes cholesterol gallstone formation. The gallstone-prone microbiota (enriched in *Desulfovibrionales*) modulated hepatic bile acid metabolism by (1) increased 7α-dehydroxylation bacteria to produce more secondary bile acids in the cecum, (2) produced H_2_S and regulated hepatic FXR-CYP7A expression, these (3) led to increasing DCA and decreasing βMCA, thereafter elevating hydrophobicity index of bile acids, inhibiting hepatic bile acid synthesis, (4) facilitating intestinal cholesterol absorption resulting in hepatic cholesterol overloading, and consequentially, (5) promoting canalicular cholesterol secretion into bile and inducing cholesterol gallstone formation.

The present study provided evidence that *Desulfovibrionales*-enriched gut microbiota contributed to gallstone formation through the mechanisms as graphically shown in Fig. 8: the gallstone-prone microbiota could (1) induce cecal bacterial 7α-dehydroxylase activities to produce more secondary bile acids; (2) produce H_2_S which could induce hepatic FXR and inhibit CYP7A1 expression and bile acid synthesis, (3) these collectively, modulate hepatic bile acid metabolism to produce more hydrophobic bile acids due to increased DCA and decreased βMCA, which increases HI of bile acids facilitating intestinal cholesterol absorption (4) resulting in hepatic cholesterol overloading, and thereafter (5) promoting canalicular cholesterol secretion into bile and gallstone formation.

The gut microbiota enriched in *Desulfovibrionales* was capable to modulate bile acid profiles to be more hydrophobic. *Desulfovibrionales* are responsible for metabolizing dietary and host-derived sulfur-containing compounds^23^. Taurine is a source of sulfur-containing substance and *Desulfovibrionale*-derived H_2_S could favor the growth of 7α-dehydroxylating bacteria growth^19^. A similar need for taurine as a growth-stimulating factor that is completely reduced to H_2_S has been demonstrated for other intestinal bacteria^24^. In line with this, we found in mice enriched in *Desulfovibrionales* (human FMT and co-housing mice), the activities of 7α-dehydroxylation were all higher. Accordingly, these mice had more secondary bile acids production as well. The other reported gut bacteria containing bile salt hydrolase activity included *Clostridium*, *Bifidobacterium*, and *Lactobacillus*^25^. Free bile acids are sequentially converted to secondary bile acids mediated by 7α-dehydroxylation reaction catalyzed by some species of *Clostridium*. However, no significant difference of species of *Clostridium* was found among groups of mice. Barely any *Bifidobacterium* and *Lactobacillus* were detected in any strain of mice under LD due to the inhibition of their growth by bile acids as DCA^26^. This is also in line with similar activities of bile salt hydrolase among groups. An increase in *Desulfovibrionales* has been described in mice fed with a high-fat diet as well^27,28^. These bacteria contain genes for sulfate reduction to reduce sulfate to H_2_S, damaging the gut barrier^29^ and producing endotoxins^30^ and pro-inflammatory cytokines such as IL6^31,32^. Furthermore, in the present study, we showed that H_2_S was capable to regulate hepatic bile acid metabolism by induction of FXR and inhibition of CYP7A1. This regulatory pathway has been proposed previously in HepG2 cells^20,21^. Here, we further collaborated the presence of such regulatory pathway in mice in vivo as well as in cell lines in vitro.

Furthermore, the gut microbiota might play a role to modulate bile acid synthesis in the liver by alleviating FXR inhibition through TβMCA, a reported FXR antagonist^33^. In single housing GS-R mice, *Cyp7a1* was less inhibited due to the presence of considerable TβMCA^33^. After human FMT or co-housing introducing *Desulfovibrionales* to GS-R mice, these mice had a similar bile acid profile with GS-S mice and hepatic *Cyp7a1* expression was reduced accordingly. The alteration of cecal and hepatic bile acid metabolism thus resulted in the elevation of HI of bile acids which could augment intestinal cholesterol absorption, hepatic cholesterol overloading, and biliary cholesterol output^34^.

Cholesterol overloading is not only due to exogenous cholesterol absorption, but de novo cholesterol synthesis as well. In GS-S mice, hepatic cholesterol de novo synthesis retained high as we observed, which is in line with the previous findings^4,35^. In human FMT or co-housing gallstone-resistant mice, expressions of key enzymes, *Hmgcr*, *Fdps*, *Sqle*, were less inhibited suggesting the maintenance of higher hepatic cholesterol synthesis in these mice even under cholesterol overloading. Furthermore, bile acid synthesis is one way to degrade cholesterol. The more inhibition of bile acid synthesis could also partly account for the more accumulation of cholesterol in the liver in mice carrying gut microbiota *Desulfovibrionale*.

Excessive hepatic cholesterol, in turn, could upregulate canalicular cholesterol transporters *Abcg5*/*g8* via activation of LXRα and enhance biliary cholesterol secretion^36^. In all mice carrying gut microbiota *Desulfovibrionale* by FMT or co-housing, the expression levels of *Abcg5*/*g8* were induced and correlated with higher biliary cholesterol molar percentage. Induction of hepatic secretion of cholesterol further collaborated in mice transplanted with species of *Desulfovibrio*. This indicated a potential activation of LXRα on *Abcg5/g8* in mice carrying *Desulfovibrio* species.

In conclusion, in this study, we demonstrated that carrying *Desulfovibronales* promoted gallstone formation. The gallstone-prone microbiota was able to induce gallstone formation by modulating bile acid hydrophobicity and promoting biliary cholesterol secretion. The results of our present study evoked our awareness of gut microbiota participating in the regulation of enterohepatic bile acid and cholesterol metabolism in promoting cholesterol gallstone formation.

## Methods

All the study protocols for research animals and human study participants were approved by the Ethical Committee at Shanghai East Hospital, Tongji University School of Medicine (EC. D (BG). 016. 02.1 (2017-027)). Written informed consent was obtained from patients who provided their feces samples.

Reagents used in the study are listed in Supplementary Table 1.

### Patient information

Feces samples were provided by patients who underwent laparoscopic cholecystectomy due to GS (GS, *n* = 80) or gallstone-free volunteers who underwent health examination (GSF, *n* = 49). Only the patients with cholesterol type of gallstone were included by classification as typical cholesterol by visual inspection of cut-surface of gallstones and when necessary, by chemical analysis in the laboratory^37,38^. All the controls were confirmed to be gallstone-free by B-type ultrasonography. Subjects who had been taken antibiotics during the past 3 months prior to sampling were excluded. Subjects with metabolic disorders, such as obesity, diabetes mellitus, hyperlipidemia, or chronic bowel disease, or chronic diarrhea, or constipation, were also excluded.

### Mice study

Male C57BL/6J mice were bought from Shanghai SLAC Laboratory Animal Company Ltd. (Shanghai, China) and AKR/J mice from Jackson Laboratory (Bar Harbor, ME, USA). Male mice (at age of 7–8 weeks) were fed with LD (containing 1.25% cholesterol and 0.5% cholic acid, synthesized by Trophic Animal Feed High-Tech Co. Ltd, Nantong, China) for 8 weeks before sacrifice. The mice were bred at the Animal Care Facility on a 12-h light/12-h dark cycle in a controlled temperature (22.5 ± 2.5 °C) and humidity (50 ± 5%) environment.

In human fecal microbiota transplantation (HuFMT) study, AKR/J mice were orally gavaged with donor human feces (dissolved in phosphate-buffered saline (PBS) 1.5 ml/200 mg of feces weight) once after depletion of gut microbes by antibiotics cocktails (containing 0.5 g/l vancomycin, 1 g/l neomycin sulfate, 1 g/l metronidazole, 1 g/l ampicillin^39^) in drinking water ad libitum for 3 weeks after weaning (at the age of 4 weeks). Donor feces were selected from the patient cohort in result 1 (Fig. 1A). The gallstone-patient donors were the 3 patients whose feces were the top 3 with enrichment of *Desulfovibrionales* as determined by 16s rRNA sequencing and GSF donors were 3 gallstone-free subjects whose feces had no detection of *Desulfovibrionales*. Feces sample from each donor was gavaged to 4 recipient mice (1 donor: 4 recipient mice). All mice were then fed with LD for 8 weeks (one mouse in the FMT from the gallstone patient donor group died after gavage).

In the co-housing study, AKR/J mice were given a cocktail of antibiotics for 3 weeks after weaning. Afterward, the AKR/J mice were co-housing with conventional C57BL/6 mice at a ratio of 1:1 for 1-week and then fed with LD for 8 weeks.

In bacteria inoculation study, *Desulfovibrio vulgaris* (ATCC-29579), *Desulfovibrio desulfuricans* (ATCC-29577), and *Desulfomonas pigra* (ATCC-29098) were purchased from ATCC, and cultured in anaerobically sterilized ATCC medium 1249 under strict anaerobic conditions overnight. C57BL/6J or AKR/J mice were depleted of gut microbes by antibiotics cocktails for 3 weeks after weaning and then oral gavaged with sterile PBS or live mixed *Desulfovibrio* (1:1:1) at a dose of 5 × 10^8^ colony forming units/100 μl in sterile PBS, respectively, three times a week for 4 weeks. Upon starting of gavage, mice were fed with LD for 8 weeks. After 4-week feeding, bile duct cannulation was performed in a subset of mice according to the procedure as described^40^. In brief, the common bile duct of mice fasted overnight was ligated and the common bile duct was cannulated with a PE-10 polyethylene catheter below the entrance of the cystic duct. The cystic duct was doubly ligated and a cholecystectomy was performed and the first-hour hepatic bile was collected.

In exogenous H_2_S study, 8-week-old C57BL/6J male mice were administered with either vehicle alone (normal saline) or vehicle containing GYY4137 (133 μmol/kg/day) (Cat No.B7458, APExBIO Technology, Houston, USA) for 7 days by intraperitoneal injection. GYY4137 is a novel H_2_S donor that could release H_2_S slowly and mimic the time course of H_2_S release in vivo^22^.

All mice were fasted 8 h before sacrifice. The blood sample was obtained by heart puncture. Liver, gallbladder, intestine, and cecal contents were collected and stored at −80 °C under analysis.

### Cell culture and experiment conditions

Mouse hepatocytes, Hepa1-6 cells, were cultured in Dulbecco’s Modified Eagle Medium (DMEM)-high glucose (Cat No. 12430054, Gibco, New York, USA) supplemented with 10% fetal bovine serum (FBS) and 1 mM sodium pyruvate (Cat No. 11360070, Gibco, New York, USA). Human hepatoma cells, HuH-7 cells, were cultured in DMEM-high glucose (Cat No. 11960044, Gibco, New York, USA) supplemented with 10% FBS, 1 mM sodium pyruvate (Cat No. 11360070, Gibco, New York, USA), and GlutaMAX™ (Cat No. 35050061, Gibco, New York, USA). Cells with 80–85% confluence were incubated with or without GYY4137 (300 or 600 μM) and GW4064 (5 μM) (Cat No. B1527, APExBIO Technology, Houston, USA).

### Analysis of biliary lipids

Total bile acids, phospholipids, and cholesterol concentration in the gallbladder or hepatic bile were measured by enzymatic methods as previously described^38,41^ using commercial purchased kits (cholesterol: Cat No. 11491458216, from Roche Diagnostics GmbH, Germany; total bile acids: Cat No. BI2672 from RANDOX, UK; and phospholipids: phospholipid LabAssay kit, Cat No. WAKO 296-63801, from Fuji Film WAKO Pure Chemical Co, Japan). The relative concentrations of biliary lipids were expressed as molar percentages of the total biliary lipids. The CSI was calculated according to Carey’s critical table^42^.

### Lipid analysis

Twenty-five microliters of serum were diluted and analyzed for cholesterol level on HITACHI automated biochemical analyzer. Lipids in liver tissue (50 mg) were extracted by chloroform:methanol solution (2/1, v/v), and total cholesterol levels were determined by enzymatic kit as previously described^43^. Serum campesterol and β-sitosterol levels were measured by gas-liquid chromatography-mass spectrometry as described^44^.

### Measurement of H_2_S level in serum

Serum H_2_S was assayed by spectrophotometry as described^45^. Briefly, 75 μl serum was reacted with 250 μl zinc acetate (1%), 425 μl distilled water, *N*,*N*-dimethyl-p-phenylenediamine sulfate in 7.2 M HCl (133 μl, 20 mM) and FeCl_3_ in 1.2 mM HCl (133 μl, 30 mM). The sample was incubated at room temperature for 10 min and then the reaction was stopped by adding 10% v/v trichloroacetic acid (250 μl). After centrifugation, the absorption of the supernatant was measured at 670 nm by spectrophotometry. NaHS (2.5–200 μM) was used as a standard.

### Bile acid analysis

Gallbladder bile, liver tissue homogenates, or cecal content were diluted with internal standard, vortexed, and purified by the sedimentation plate. Samples were then lyophilized and dissolved with 25% acetonitrile. After centrifugation, the supernatants were collected for measuring the levels of various bile acids. Bile acids profiles were analyzed on an Acquity UPLC system coupled to a Waters Xevo TQ-S MS (Waters, Manchester, UK), equipped with a C18 reverse-phase column with 1.7 mm particle size (Waters Corp., Milford, MA, USA). Analytes were detected by electrospray ionization and quantified by internal standard methods as described^46^. The HI of bile acids in gallbladder bile was calculated as described^47^.

### Gut flora activity assays

Activities of the gut flora were measured according to the procedure as described^48,49^. One gram of cecal contents of each mouse was dissolved in 3 ml sterilized solution A (containing 20 % glycerol and 1.8 % sodium chloride) which was diluted in polyPeptone Yeast Extract Medium (PY culture medium) and then cultured at 37 °C and 200 rpm for 12 h to allow anaerobic bacteria to proliferate. Afterward, 200 μl tauro-cholic acid (200 μg/ml) was added in 1 ml the mixture and incubated for another 1 h at 37 °C in the anaerobic chambers and the reaction was stopped by 100 μl of 1 M HCl. The generation of cholic acid and deoxycholic acid were measured by Acquity UPLC system coupled to a Waters Xevo TQ-S MS for the calculation of gut flora activities.

### Microbiota analysis

Total genomic bacterial DNA was extracted from cecal samples using the QIAamp Fast DNA Stool Mini Kit (Qiagen, Germany) following the manufacturer’s instructions. PCR amplification of bacteria DNA was performed as previously described^46^. Amplicons were extracted from 2% agarose gel, purified by the AxyPrep DNA gel extraction kit (Axygen Biosciences, Union City, CA, USA), and quantified by QuantiFluorTM-ST (Promega, Madison, WI, USA) according to the manufacturer’s protocols. Then purified amplicons were pooled in equimolar amounts and paired-end sequenced on an Illumina MiSeq platform following standard instructions by the commercial service of Genergy Biotechnology Co. Ltd. (Shanghai, China).

Raw Illumina fasta files were de-multiplexed, quality filtered, and analyzed using the QIIME software according to standardized criteria as described^50^. Operational taxonomic units (OTUs; 97% sequence similarity) were clustered using the UPARSE software (version 7.1, http://drive5.com/uparse/), and chimeric sequences were identified and removed using the UCHIME program. The phylogenetic affiliation of each 16S rRNA gene sequence was analyzed using the Ribosomal Database Project Classifier tool (version 11.1, http://rdp.cme.msu.edu/) against the SILVA (SSU115) 16S rRNA database (http://www.arb-silva.de) using a confidence threshold of 70%. Alpha diversity was used to describe the microbial richness, diversity, and evenness within samples with four parameters: two richness estimators (Chao1 and the abundance-based coverage estimator) and two diversity indices (Shannon and Simpson indices). Jackknifed beta diversity analysis (between-sample diversity comparisons) was calculated using weighted and unweighted unifrac distances between samples, and principal coordinates were also computed to compress dimensionality into two-dimensional principal coordinate analysis plots. Observed species alpha rarefaction of filtered OTU tables was also performed to confirm that the sequence coverage was adequate to capture the species diversity observed in all samples. Identification of microbial genes involved in BA biotransformation was performed using methods as reported^51^.

### RNA-sequencing

Total RNA was extracted using TRIzol reagent (Invitrogen, Carlsbad, CA, USA). RNA quality was examined by gel electrophoresis and with a Nanodrop spectrophotometer (Thermo, Waltham, MA, USA). cDNA library generation with the TruSeqRNA Sample Preparation Kit (Illumina, San Diego, CA, USA). Clusters were generated with the TruSeq SR Cluster Kit v2 according to the reagent preparation guide.

The RNA sequencing was performed using the HiSeq X Ten platform by the commercial service of Genergy Biotechnology Co. Ltd. (Shanghai, China). The raw data was handled by Perl and data quality was checked by FastQC 0.11.2 (http://www.bioinformatics.babraham.ac.uk/projects/fastqc/). RNA-Seq reads were aligned to the reference data downloaded from UCSC (version hg19), and the RPKM method was utilized to normalize the reads that exclusively mapped to a gene, to quantify the transcript levels^52^.

### Western blot analysis

Total protein from frozen livers or cultured cells were extracted and used with western immunoblotting as described previously^53^. The following antibodies were used for western blot: anti-FXR (1:1000, Abcam, ab235094), anti-CYP7A1 (1:1000, Abcam, ab65596), anti-CYP8B1 (1:1000, Abcam, ab191910), anti-GAPDH (1:1000, Proteintech, 60004-1-Ig). Images were quantified using the Image Pro Plus 6.0 software.

### Quantitative real-time PCR

Power Mix Sybr Green Master Mix (Applied Biosystems) was used for quantitative real-time PCR to determine the target gene expressions. The relative mRNA expression was calculated with the ΔΔCt method using GAPDH as the internal control as described^43^. Primer sequences are listed in Supplementary Table 1.

### Statistics

Quantitative data are expressed as mean ± SEM and qualitative data as a ratio. The difference between the two groups was compared by *t* test and among multiple groups by analysis of variance followed by least significant difference test for post hoc analysis between the groups. Two-sided *P* < 0.05 was regarded as statistical significance. All the statistics were performed using the SPSS 12.0 software.

### Reporting summary

Further information on research design is available in the Nature Research Reporting Summary linked to this article.

## Supplementary information

Supplementary Information

Reporting Summary

## Data Availability

All the data are available upon request. The raw data of 16s rRNA sequencing generated in this study has been deposited at NCBI SRA BioProject (accession no: PRJNA773136) and that of RNA-seq at EMBL-EBI (https://www.ebi.ac.uk/arrayexpress/experiments/E-MTAB-8550). Source data are provided with this paper.

## Source data

Source Data

## Code availability

Scripts for plots under python are available at the website https://github.com/zhajia2019/Python_Scripts.
