# Supplementary material for: Gut microbiota promotes cholesterol gallstone formation by modulating bile acid composition and biliary cholesterol secretion
Source: Nat Commun. 2022 Jan 11;13:252. doi: 10.1038/s41467-021-27758-8 (PMC8752841; doi:10.1038/s41467-021-27758-8)
Supplement: Supplementary file 1 — Supplementary Information [file 41467_2021_27758_MOESM1_ESM.pdf]

Supplementary Information

Gut microbiota promoted cholesterol gallstone formation by modulating bile acid composition and biliary cholesterol secretion

Hai Hu, Wentao Shao, Qian Liu, Ning Liu, Qihan Wang, Jin Xu, Xin Zhang,

Zhenkun Weng, Qifan Lu, Long Jiao, Chaobo Chen, Haidong Sun,

Xiaoping Zhang, Zhaoyan Jiang, Aihua Gu

Supplementary FigureS1-5

Supplementary TableS1-3

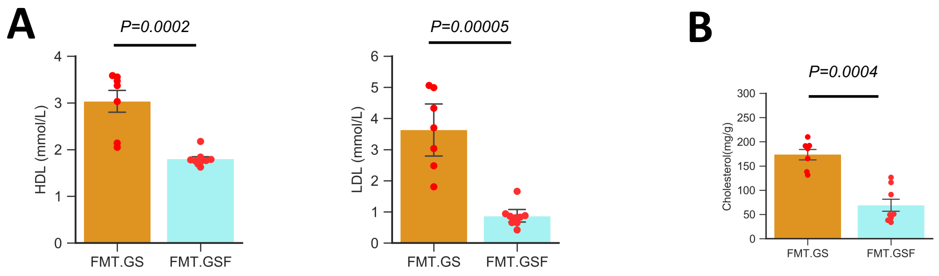

Figure S1

Figure S1. A. Serum cholesterol in high density lipoprotein (HDL) and low density lipoprotein (LDL) (FMT.GS: n=7 and FMT.GSF: n=9). Data were expressed as means $\pm$ SEM. Statistics was performed by 2-sided t-test. **B.** Hepatic cholesterol levels between groups (FMT.GS: n=7 and FMT.GSF: n=9). Data were expressed as means $\pm$ SEM. Statistics was performed by 2-sided t-test. "FMT.GS": mice receiving fecal transplantation from patient with cholesterol gallstone; "FMT.GSF": mice receiving fecal transplantation from gallstone-free controls. Source data are provided as a Source Data file.

A

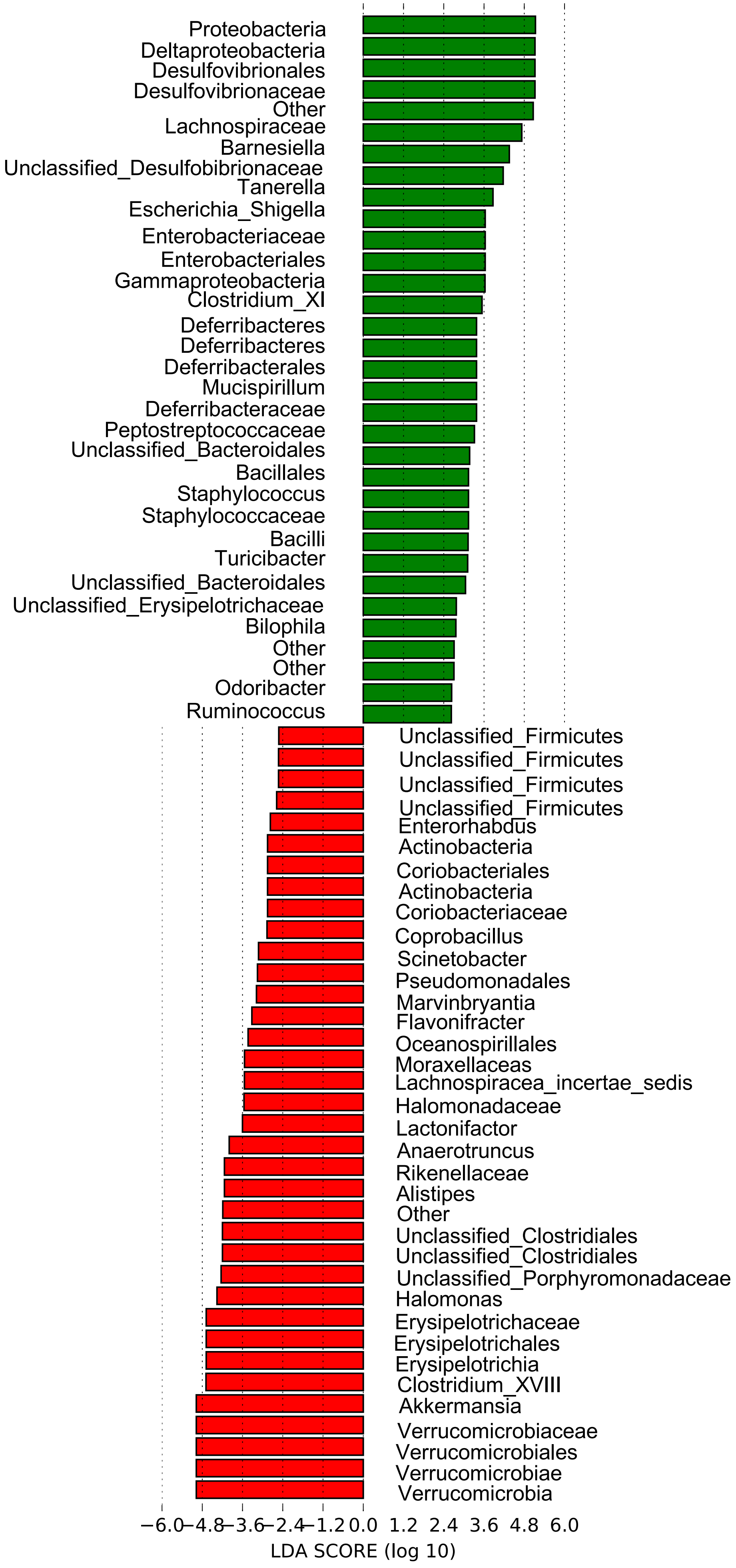

Proteobacteria
Deltaproteobacteria
Desulfovibrionales
Desulfovibrionaceae
Other
Lachnospiraceae
Barnesiella
Unclassified_Desulfobibrionaceae
Tanerella
Escherichia_Shigella
Enterobacteriaceae
Enterobacteriales
Gammaproteobacteria
Clostridium_XI
Deferribacteres
Deferribacteres
Deferribacterales
Mucispirillum
Deferribacteraceae
Peptostreptococcaceae
Unclassified_Bacteroidales
Bacillales
Staphylococcus
Staphylococcaceae
Bacilli
Turicibacter
Unclassified_Bacteroidales
Unclassified_Erysipelotrichaceae
Bilophila
Other
Other
Odoribacter
Ruminococcus
Unclassified_Firmicutes
Unclassified_Firmicutes
Unclassified_Firmicutes
Unclassified_Firmicutes
Enterorhabdus
Actinobacteria
Coriobacteriales
Actinobacteria
Coriobacteriaceae
Coprobacillus
Scinetobacter
Pseudomonadales
Marvinbryantia
Flavonifracter
Oceanospirillales
Moraxellaceas
Lachnospiracea_incertae_sedis
Halomonadaceae
Lactonifactor
Anaerotruncus
Rikenellaceae
Alistipes
Other
Unclassified_Clostridiales
Unclassified_Clostridiales
Unclassified_Porphyromonadaceae
Halomonas
Erysipelotrichaceae
Erysipelotrichales
Erysipelotrichia
Clostridium_XVIII
Akkermansia
Verrucomicrobiaceae
Verrucomicrobiales
Verrucomicrobiae
Verrucomicrobia
−6.0 −4.8 −3.6 −2.4 −1.2 0.0 1.2 2.4 3.6 4.8 6.0
LDA SCORE (log 10)

Figure S2

Figure S2. LefSe analysis of gut microbiota between C57BL/6J and AKR/J mice under lithogenic diet. Specific different phylotypes of gut bacteria between strains of mice. LDA scores were computed for features at the OTU level (n=5 mice/group). Source data are provided as a Source Data file.

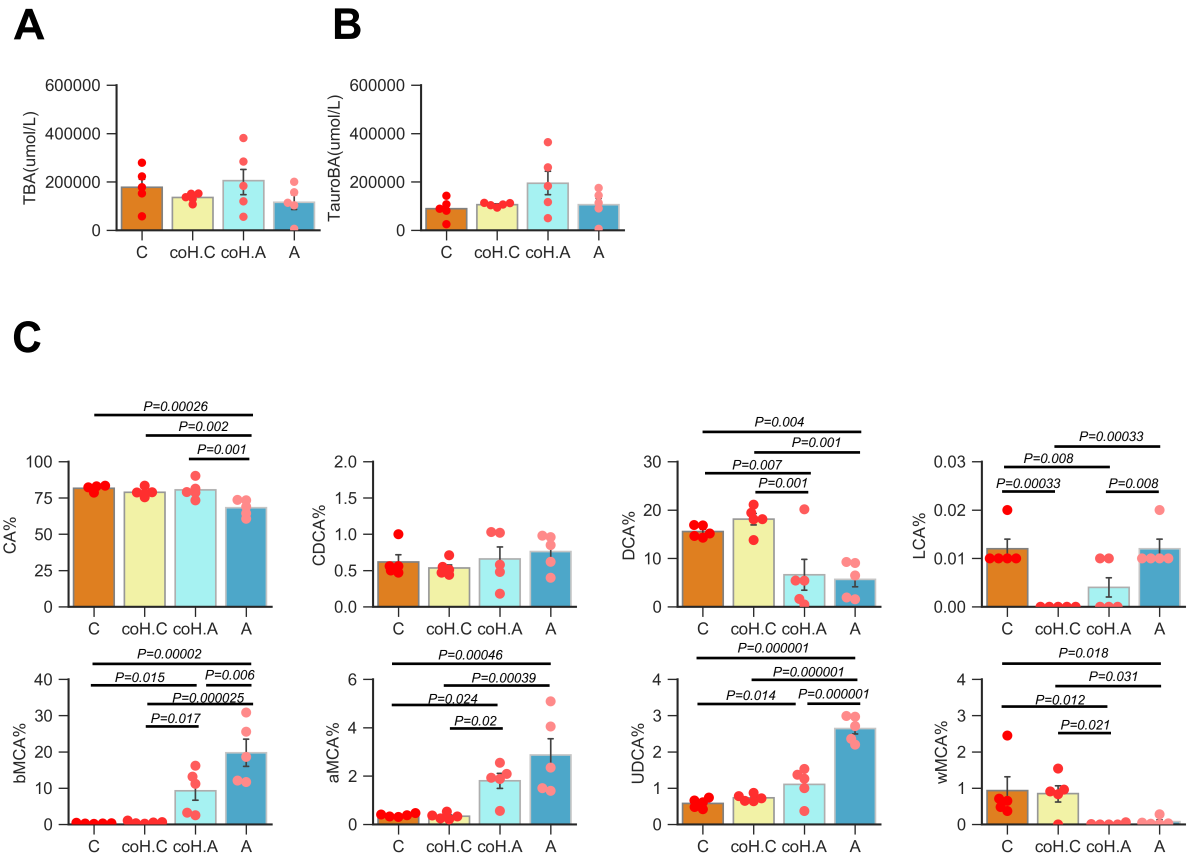

Figure S3

Figure S3. Comparison of bile acid composition in gallbladder bile from single housing C57BL/6J and AKR/J mice and the co-housing mice.

A. Total bile acids (n=5 mice/group). Data were expressed as means ± SEM. Statistics was performed by 2-sided ANOVA with LSD post-hoc analysis between groups. **B**. tauro-conjugated bile acids amounts (n=5 mice/group). Data were expressed as means ± SEM. Statistics was performed by 2-sided ANOVA with LSD post-hoc analysis between groups. **C**. Bile acid compositions (n=5 mice/group). Data were expressed as means ± SEM. Statistics was performed by 2-sided ANOVA with LSD post-hoc analysis between groups. “C”: C57BL/6J, “A”: AKR/J, “coH.C”: C57BL/6J co-housing with AKR/J and “coH.A”: AKR/J co-housing with C57BL/6J. Source data are provided as a Source Data file.

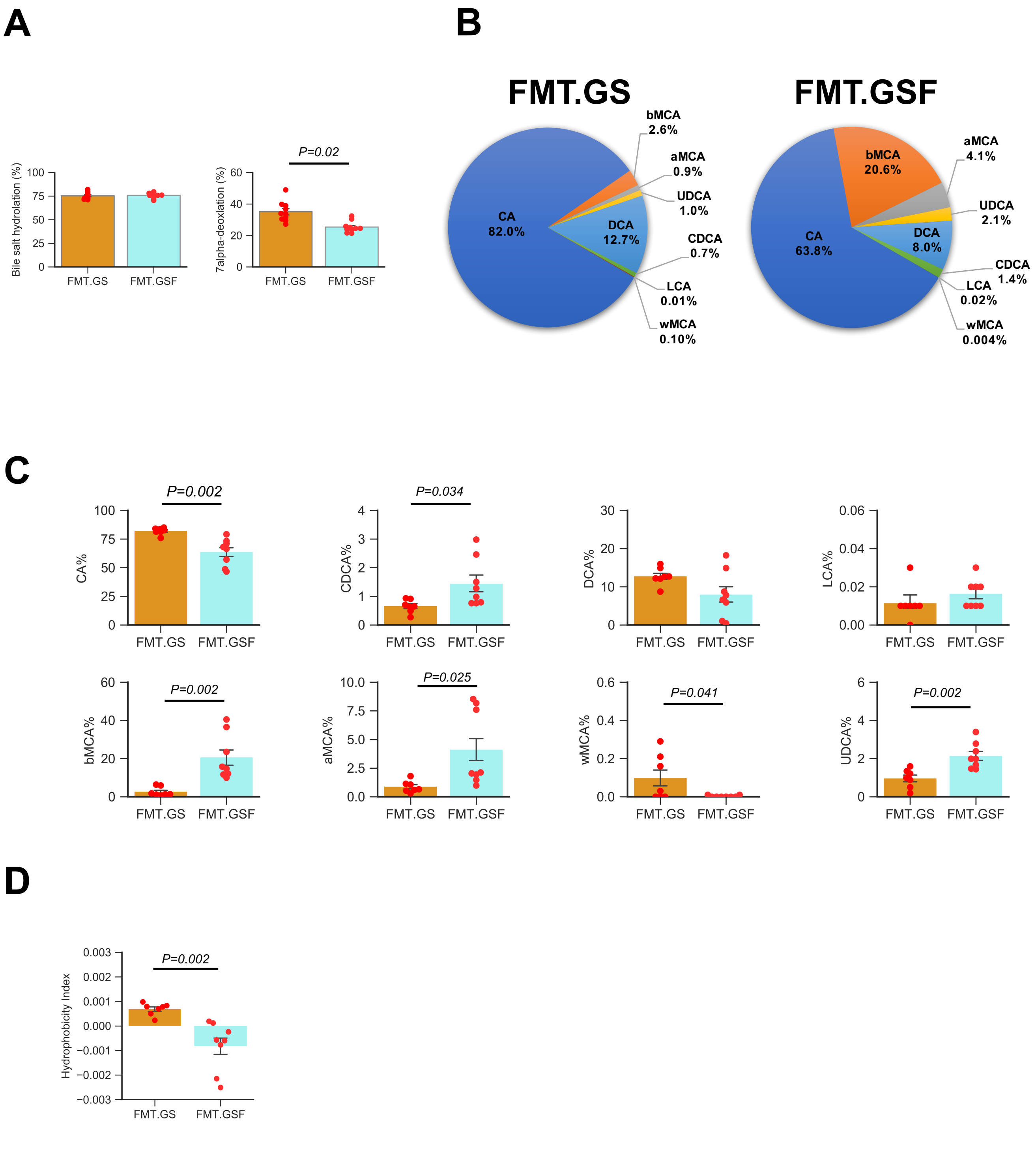

A
Bile salt hydrolation (%)
100
75
50
25
0
FMT.GS
FMT.GSF
7alpha-deoxlation (%)
60
40
20
0
P=0.02
B
FMT.GS
FMT.GSF
bMCA 2.6%
aMCA 0.9%
UDCA 1.0%
CDCA 0.7%
LCA 0.01%
wMCA 0.10%
CA 82.0%
DCA 12.7%
bMCA 20.6%
aMCA 4.1%
UDCA 2.1%
DCA 8.0%
CA 63.8%
CDCA 1.4%
LCA 0.02%
wMCA 0.004%
C
CA%
P=0.002
CDCA%
P=0.034
DCA%
LCA%
bMCA%
P=0.002
aMCA%
P=0.025
wMCA%
P=0.041
UDCA%
P=0.002
D
Hydrophobicity Index
P=0.002
0.003
0.002
0.001
0.000
−0.001
−0.002
−0.003
FMT.GS
FMT.GSF

Figure S4

Figure S4. Differences in cecal bacterial activities of bile acid metabolism and comparison of bile acid composition in gallbladder bile from gallstone-resistant mice AKR/J receiving fecal microbiota transplantation (FMT) from cholesterol gallstone patients (GS) or gallstone-free controls (GSF).

A. Bacterial bile salt hydrolase and 7a-dehydroxylation activities in cecum contents (FMT.GS: n=7 and FMT.GSF: n=9). Data were expressed as means ± SEM. Statistics was performed by 2-sided t-test. **B**. Pie plot and **C**. bar plot of bile acid compositions (FMT.GS: n=7 and FMT.GSF: n=9). Data were expressed as means ± SEM. Statistics was performed by 2-sided t-test**. D.** Bile acid hydrophobicity index (FMT.GS: n=7 and FMT.GSF: n=9). Data were expressed as means ± SEM. Statistics was performed by 2-sided t-test. “FMT.GS”: FMT from donor patients with cholesterol gallstone to AKR/J mice; “FMT.GSF”: FMT from gallstone-free controls to AKR/J mice. Source data are provided as a Source Data file.

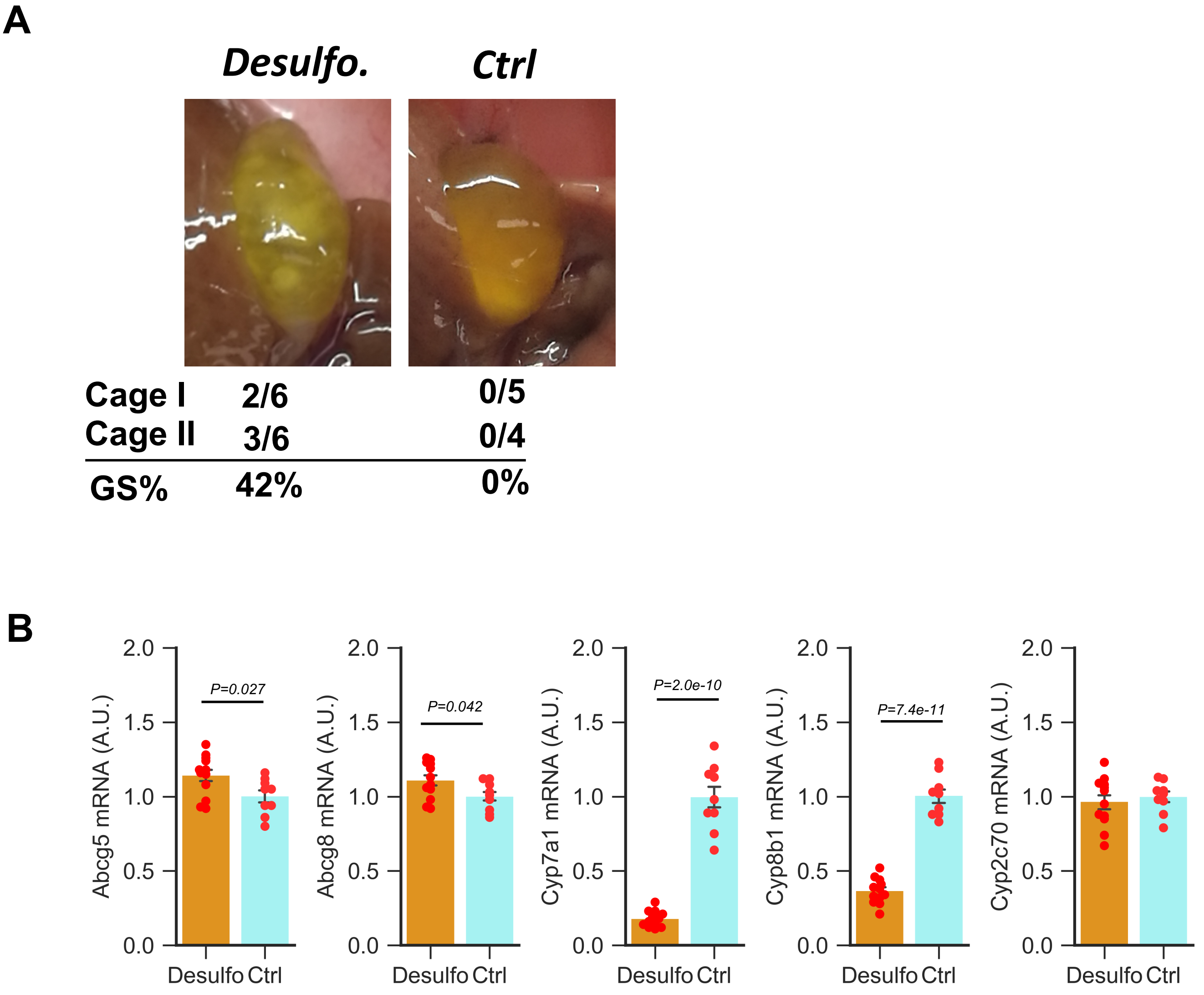

Figure S5

Figure S5. Species of *Desulfovibro* promoted gallstone formation in gallstone-resistant mice AKR/J. A. Incidence of gallstone between groups. Gallstone formed in 42% (5/12) of the AKR/J mice after *Desulfovibrio* species transplantation and none form 0%(0/9) in the control mice. **B.** Comparison of hepatic gene expression between groups (Desulfo group: n=12, Ctrl group: n=9). Data were expressed as means ± SEM. Statistics was performed by 2-sided t-test. "*Desulfo*": AKR/J mice receiving *Desulfovibrio* species. "Ctrl": AKR/J mice receiving saline. Source data are provided as a Source Data file.

TableS1 Reagents used in the study

Reaget / Kit name	Cat No	Venders
Vancomycin hydrochloride	V2002	Sigma Aldrich, USA
Neomycin trisulfate salt hydrate	N6386	Sigma Aldrich, USA
Ampicillin	A9393	Sigma Aldrich, USA
Metronidazole	M1547	Sigma Aldrich, USA
Bile acid standards	-	Sigma Aldrich, USA
d4 or d5-labelled bile acids	-	Toronto Research Chemicals, Canada
Cholesterol assay (CHOD-PAP)	11491458	Roche/Hitachi, Germany
Total bile acids assay	B12672	RANDOX, UK
LabAssay Phospholipid	296-63801	WAKO, Japan
TRIzol™ Reagent	15596018	Invitrogen, USA
High Capacity cDNA Reverse Transcription Kit	4368814	Applied Biosystems, USA
Sybr Green PowerUp™ SYBR™ Green Master Mix	A25777	Applied Biosystems, USA
QIAamp Fast DNA Stool Mini Kit	51604	Qiagen, Germany
TruSeq® RNA LT Sample Prep Kit v2	RS-122-2001	Illumina, USA
TruSeq PE Cluster Kit v3 - cBot – HS	PE-401-3001	Illumina, USA
TruSeq SBS Kit v3 - HS (200-cycles)	FC-401-3001	Illumina, USA
AmpureBeads	A63881	Beckman, USA
Anti-Bile Acid Receptor NR1H4(FXR) antibody	ab235094	Abcam
Anti-CYP7A1 antibody	ab65596	Abcam
Anti-CYP8B1 antibody	ab191910	Abcam
Anti-GAPDH antibody	5174	Cell Signaling Technology

Table S2 Primer sequences for real-time PCR

Gene	FP	RP
Cyclophilin A	GAT GAG AAC TTC ATC CTA AAG CAT ACA	TCA GTC TTG GCA GTG CAG ATA AA
Hmgcr	TGA TTG GAG TTG GCA CCA T	TGG CCA ACA CTG ACA TGC
Ldlr	GGA TGG CTA TAC CTA CCC CTC AA	CAC ATC GTC CTC CAG GCT G
Srbi	CGG GAG CGT GGA CCC TAT GT	ACA CGG TGT CGT TGT CAT TGA
Cyp7a1	AGC AAC TAA ACA ACC TGC CAG TAC TA	GTC CGG ATA TTC AAG GAT GCA
CYP27	GCC TTG CAC AAG GAA GTG ACT	CGC AGG GTC TCC TTA ATC ACA
Cy08b	GAA CTC AAC CAG GCC ATG CT	GGC ACC CAG ACT CGA ACC T
Abca1	CCT GCT AAA ATA CCG GCA AGG	AGT AAC CCG TTC CCA ACT GGT
Abcb11/Bsep	CAA TAG ACA GGC AAC CCG TCA	GTG GAA CTC AAT TTC GCC CTT
Abcb4/Mdr2	GCA GCG AGA AAC GGA ACA G	GGT TGC TGA TGC TGC CTA GTT
Abcg1	GCT GAA GAG GAC TCC GCC T	GAG GAT GCA GAA CTG GGT GAG
Abcg5	AAT GCT GTG AAT CTG TTT CCC A	CCA CTT ATG ATA CAG GCC ATC CT
Abcg8	TGC CCA CCT TCC ACA TGT C	ATG AAG CCG GCA GTA AGG TAG A
Fgf15	GGT CGC TCT GAA GAC GAT TG	CGC GCT CAT GCA GAG GTA
Asbt	TGG CGA CAT GGA CCT CAG T	GAG GCA AAG AGG CAT CAT TCC
Ibabp	CAGGAGACGTGATTGAAAGGG	GCCCCCAGAGTAAGACTGGG
Fgf15	GGT CGC TCT GAA GAC GAT TG	CGC GCT CAT GCA GAG GTA
Ost a	TGTTCCAGGTGCTTGTCATCC	CCACTGTTAGCCAAGATGGAGAA
Ost b	GATGCGGCTCCTTGGAATTA	GGAGGAACATGCTTGTCATGAC

TableS3 Softwares used in the study

Software	Version	Company or website
SPSS	V21.0	IBM, USA
Python	V3.7.2	https://www.python.org/downloads/mac-osx/
R software	V3.4.3	https://www.r-project.org
Anaconda-Navigator	V1.8.1	https://www.anaconda.com/download/#macos
Excel	V15.28	Microsoft, USA
