# Supplementary material for: Gut microbiota promotes cholesterol gallstone formation by modulating bile acid composition and biliary cholesterol secretion
Source: Nat Commun. 2022 Jan 11;13:252. doi: 10.1038/s41467-021-27758-8 (PMC8752841; doi:10.1038/s41467-021-27758-8)
Supplement: Supplementary file 2 — Reporting Summary [file 41467_2021_27758_MOESM2_ESM.pdf]

nature portfolio

Corresponding author(s): Xiaoping Zhang, Zhaoyan Jiang, Aihua Gu

Last updated by author(s): Nov 7, 2021

Reporting Summary

Nature Portfolio wishes to improve the reproducibility of the work that we publish. This form provides structure for consistency and transparency in reporting. For further information on Nature Portfolio policies, see our Editorial Policies and the Editorial Policy Checklist.

Statistics

For all statistical analyses, confirm that the following items are present in the figure legend, table legend, main text, or Methods section.

n/a	Confirmed	
☐	☒	The exact sample size (n) for each experimental group/condition, given as a discrete number and unit of measurement
☐	☒	A statement on whether measurements were taken from distinct samples or whether the same sample was measured repeatedly
☐	☒	The statistical test(s) used AND whether they are one- or two-sided<br>*Only common tests should be described solely by name; describe more complex techniques in the Methods section.*
☒	☐	A description of all covariates tested
☐	☒	A description of any assumptions or corrections, such as tests of normality and adjustment for multiple comparisons
☐	☒	A full description of the statistical parameters including central tendency (e.g. means) or other basic estimates (e.g. regression coefficient) AND variation (e.g. standard deviation) or associated estimates of uncertainty (e.g. confidence intervals)
☐	☒	For null hypothesis testing, the test statistic (e.g. F, t, r) with confidence intervals, effect sizes, degrees of freedom and P value noted<br>*Give P values as exact values whenever suitable.*
☒	☐	For Bayesian analysis, information on the choice of priors and Markov chain Monte Carlo settings
☒	☐	For hierarchical and complex designs, identification of the appropriate level for tests and full reporting of outcomes
☒	☐	Estimates of effect sizes (e.g. Cohen's d, Pearson's r), indicating how they were calculated

Our web collection on statistics for biologists contains articles on many of the points above.

Software and code

Policy information about availability of computer code

Data collection	No software was used for data collection.
Data analysis	Statistics were analyzed using SPSS12.0 software. Python 3.6 based on Anaconda platform (https://www.anaconda.com/products/individual) or microsoft excel was used to generate plots. Raw Illumina fasta files were processed and analyzed using the QIIME software (http://qiime.org). Analysis for 16sRNA sequencing and RNA-seq data were performed using R software (https://www.r-project.org).

For manuscripts utilizing custom algorithms or software that are central to the research but not yet described in published literature, software must be made available to editors and reviewers. We strongly encourage code deposition in a community repository (e.g. GitHub). See the Nature Portfolio guidelines for submitting code & software for further information.

Data

Policy information about availability of data

All manuscripts must include a data availability statement. This statement should provide the following information, where applicable:

- Accession codes, unique identifiers, or web links for publicly available datasets
- A description of any restrictions on data availability
- For clinical datasets or third party data, please ensure that the statement adheres to our policy

Source data are provided as s Source Data File with this paper. All other data are also available upon request. The raw data of 16s rRNA sequencing generated in this study has been deposited at NCBI SRA BioProject (accession no: PRJNA773136, https://www.ncbi.nlm.nih.gov/bioproject/PRJNA773136/) and that of RNA-seq at EMBL-EBI (https://www.ebi.ac.uk/arrayexpress/experiments/E-MTAB-8550).

Field-specific reporting

Please select the one below that is the best fit for your research. If you are not sure, read the appropriate sections before making your selection.

- [x] Life sciences
- [ ] Behavioural & social sciences
- [ ] Ecological, evolutionary & environmental sciences

For a reference copy of the document with all sections, see nature.com/documents/nr-reporting-summary-flat.pdf

Life sciences study design

All studies must disclose on these points even when the disclosure is negative.

Sample size	Sample sizes were estimated based on previous experiences used for cholesterol gallstone mouse model (J Lipid Res 1999, 40: 2080, J Hep 2001, 35: 444, BMC Gastroenterology 2017, 17: 74).
Data exclusions	All data collected were used in the analysis.
Replication	Number of mice (n) represented individual mice used in the statistics as described in the figure legends. Cell experiments were performed for 3 independent repetitions.
Randomization	Mice were randomly allocated into groups before starting the experiments.
Blinding	Operators in charge of the sample analysis were blind to the experimental design.

Reporting for specific materials, systems and methods

We require information from authors about some types of materials, experimental systems and methods used in many studies. Here, indicate whether each material, system or method listed is relevant to your study. If you are not sure if a list item applies to your research, read the appropriate section before selecting a response.

Materials & experimental systems

n/a	Involved in the study	
☐	☒	Antibodies
☐	☒	Eukaryotic cell lines
☒	☐	Palaeontology and archaeology
☐	☒	Animals and other organisms
☐	☒	Human research participants
☒	☐	Clinical data
☒	☐	Dual use research of concern

Methods

n/a	Involved in the study	
☒	☐	ChIP-seq
☒	☐	Flow cytometry
☒	☐	MRI-based neuroimaging

Antibodies

Antibodies used	anti-FXR (Abcam, ab235094), anti-CYP7A1 (Abcam, ab65596), anti-CYP8B1 (Abcam, ab191910), anti-GAPDH (Proteintech, 60004-1-Ig) were used in Western Blotting.
Validation	Anti-FXR antibody (ab235094) has been validated in HEK-293 whole cell lysate and used in publications (PubMed: 33977967, 34149876, 32292349, 32116693). Anti-CYP7A1 antibody (ab65596) has been used in publications (PubMed: 32553670, 34149876, 33144503). Anti-CYP8B1 antibody (ab191910) has been validated in HEK293T, RAW 264.7 and H9C2 whole cell lysates and has been used in publication (PubMed: 33679408, 33754066, 31672964). Anti-GAPDH (60004-1-Ig) has been validated in various cell lysates. See also descriptions on manufactory's website.

Eukaryotic cell lines

Policy information about cell lines

Cell line source(s)	Hepa1-6 (CRL-1830) cell line was provided by ATCC. HuH7 (SCSP-526) was provide by National Collection of Authenticated Cell Cultures (China).
Authentication	The cell lines were used between passage number 3-25 to avoid changes in the cell line characteristics. No authentication was performed for these cells.
Mycoplasma contamination	All cell lines were routinely checked for micoplasma infections with negative results.
Commonly misidentified lines (See ICLAC register)	No commonly misidentified cell lines were used in this study.

Animals and other organisms

Policy information about studies involving animals; ARRIVE guidelines recommended for reporting animal research

Laboratory animals	C57BL/6J mice were bought from Shanghai SLAC Laboratory Animal Company Ltd. (Shanghai, China) and AKR/J mice from Jackson Laboratory (Bar Harbor, ME, USA). In the only lithogenic diet feed experiment, mice age 7-8 weeks were used. In the FMT, cohousing experiments, mice were used after weening.
Wild animals	No wild animal was used in the study.
Field-collected samples	No field-collected samples were used in the study.
Ethics oversight	All the study protocols in animal and human were approved by the Ethical Committee at Shanghai East Hospital, Tongji University School of Medicine (EC. D (BG). 016. 02.1 (2017-027)).

Note that full information on the approval of the study protocol must also be provided in the manuscript.

Human research participants

Policy information about studies involving human research participants

Population characteristics	Feces sample were provided by patients underwent laparoscopic cholecystectomy due to gallstone disease or gallstone-free volunteers who underwent health examination. All the controls were confirmed to be gallstone-free by B-type ultrasonography. Subjects who had been taken antibiotics during the past 3 months prior to sampling were excluded. Subjects with metabolic disorders such as obesity, diabetes mellitus, hyperlipidemia, or chronic bowel disease, or chronic diarrhea or constipation, were also excluded.
Recruitment	Gallstone patients were patients underwent laparoscopic cholecystectomy. Only the patients with cholesterol type of gallstone were included by classification as typical cholesterol by visual inspection of cut-surface of gallstones and when necessary, by chemical analysis in the laboratory. Control were gallstone-free subjects ocnfirmed by B-type untrasonography during health examination.
Ethics oversight	Collection of human feces were approved by the Ethical Committee at Shanghai East Hospital, Tongji University School of Medicine (EC. D (BG). 016. 02.1 (2017-027)). Inform consent was obtained from patients who provided their feces samples.

Note that full information on the approval of the study protocol must also be provided in the manuscript.
